# Variation of the Neutrophil-to-Lymphocyte Ratio and Mean Platelet Volume in a Multicenter Study of Critically Ill Patients with COVID-19 Living at Different Altitudes

**DOI:** 10.3390/medsci14030363

**Published:** 2026-06-30

**Authors:** Pablo Andrés Vélez-Páez, Jorge Luis Vélez-Páez, Iván Best, Luis Arturo Herrera-León, Pedro René-Torres, Jorge Vasconez-Gonzalez, Juan S. Izquierdo-Condoy, Esteban Ortiz-Prado

**Affiliations:** 1 Facultad de Ciencias Médicas, Universidad Central del Ecuador, Quito 170403, Ecuador; 2 Unidad de Terapia Intensiva, Hospital de Especialidades Quito N° 1 de la Policía Nacional, Quito 170525, Ecuador; 3 Unidad de Terapia Intensiva, Centro de Investigación Clínica, Hospital Pablo Arturo Suárez, Quito 170315, Ecuador; 4 Carrera de Medicina, Facultad de Ciencias de la Salud, Universidad San Ignacio de Loyola, Lima 15024, Peru; 5 Hospital General Ibarra, Ibarra 100104, Ecuador; 6 Facultad de Medicina, Universidad Técnica del Norte, Ibarra 100150, Ecuador; 7 Empresa Pública de Salud Universitaria del Norte, Hospital Universitario, Ibarra 100101, Ecuador; 8 One Health Research Group, Universidad de las Americas, Quito 170124, Ecuador

**Keywords:** SARS-CoV-2, COVID-19, neutrophil-to-lymphocyte ratio, platelet volume, high altitude, low altitude

## Abstract

Background: Evidence on the prognostic utility of biological markers in critically ill patients with COVID-19 across different geographic altitudes remains limited. Objectives: The aim of this study was to determine the prognostic value of inflammatory and hematological markers, particularly the neutrophil-to-lymphocyte ratio (NLR) and mean platelet volume (MPV), for mortality risk in critically ill patients with COVID-19 receiving invasive mechanical ventilation and living at different geographic altitudes. Methods: A multicenter retrospective cohort study was conducted using secondary data from a clinical database. A total of 362 critically ill patients with COVID-19 admitted to intensive care units (ICUs) between April 2020 and March 2022 were included. Patients were treated at three hospitals in Ecuador located at different altitudes: sea level (4 m above sea level [masl]), moderate altitude (2200 masl), and high altitude (2850 masl). Clinical, laboratory, and outcome data were obtained from electronic medical records. Results: The mean age was 54.44 years, and 71.27% of patients were male. Post hoc, altitude-stratified NLR and MPV thresholds were associated with increased odds of ICU mortality: at sea level, NLR ≥ 12.50 and MPV ≥ 8.80 fL; at moderate altitude, NLR ≥ 11.50 and MPV ≥ 9.80 fL; and at high altitude, NLR ≥ 16.30 and MPV ≥ 9.00 fL. Conclusions: In mechanically ventilated patients with critical COVID-19, NLR and MPV values above post hoc, altitude-stratified cutoff points were associated with higher ICU mortality. However, because each altitude stratum corresponded to a different hospital and these thresholds were not ROC-derived or internally/externally validated, these findings should be interpreted as exploratory and hypothesis-generating.

## 1. Introduction

The COVID-19 pandemic led to significant changes in intensive care unit (ICU) patient management, with ICU admissions rising rapidly, particularly during the early phase of the outbreak [1,2]. A substantial proportion of patients required intubation due to severe respiratory deterioration. Accordingly, the high morbidity and mortality associated with COVID-19 have been largely linked to the development of acute respiratory distress syndrome (ARDS) [2,3].

Differences in morbidity and mortality among patients affected by this infection have been attributed to various factors, including socioeconomic status, the burden of chronic diseases, and access to adequate healthcare [4]. In addition, residence at high altitude has been associated with lower COVID-19 attack and mortality rates, suggesting that geographic altitude may serve as a potential modulator of clinical outcomes in this population [5].

Several studies have explored the relationship between altitude and COVID-19-related outcomes. Karsli et al. reported that high-altitude adaptations, such as increased lung capacity and epigenetic changes, may mitigate COVID-19 severity [6]. Jibaja et al. found that patients treated at high altitude had a 74% higher likelihood of ICU survival/discharge and a 35% higher likelihood of hospital survival/discharge than those treated at sea level [7]. In addition, study conducted in ICUs in Ecuador showed that residence at high altitude was associated with improved survival, particularly among patients without comorbidities [4].

On the other hand, biomarkers may be useful for improving the assessment of disease severity and guiding the therapeutic management of patients with COVID-19 [8]. A meta-analysis reported that mean platelet volume (MPV) was associated with disease severity and mortality in patients with COVID-19. In addition, higher neutrophil-to-lymphocyte ratio (NLR) values at ICU admission have been linked to worse clinical outcomes in critically ill patients with COVID-19 [9,10]. Despite these findings, evidence regarding the prognostic value of different biological markers in severely ill patients with COVID-19 residing at high altitude remains limited [8]. 

The aim of this study was to determine the prognostic value of inflammatory and hematological markers, such as NLR and MPV, for mortality risk in critically ill patients with COVID-19 receiving invasive mechanical ventilation and residing at different geographic altitudes.

## 2. Materials and Methods

### 2.1. Study Design

A multicenter, retrospective cohort study was conducted using secondary data analysis obtained from a clinical database.

### 2.2. Setting

The study was carried out in Ecuador, a country located in northwestern South America and characterized by marked geographic diversity, including coastal and Andean regions with substantial differences in altitude [11]. Ecuador’s healthcare system is composed of the public sector, the social security system, and the private sector, all of which contribute to the provision of hospital care [12].

### 2.3. Population and Sample

The study sample comprised 362 critically ill patients with COVID-19 admitted to the ICU between April 2020 and March 2022 at three hospitals located at different altitudes in Ecuador: 120 patients from Hospital Los Ceibos in Guayaquil, Ecuador, located at sea level (4 m above sea level [masl]); 121 patients from Hospital General in Ibarra, Ecuador, located at 2200 masl; and 121 patients from Hospital Pablo Arturo Suárez in Quito, Ecuador, located at 2850 masl. These institutions provide intensive care services for adult patients with severe COVID-19 and allow the evaluation of outcomes across distinct altitude settings.

### 2.4. Inclusion Criteria

Patients of both sexes aged ≥18 years who were admitted to the ICU with a confirmed diagnosis of COVID-19 by RT-PCR, antigen testing, or a suggestive tomographic pattern (CO-RADS 4 or 5), and who required invasive mechanical ventilation, were included.

### 2.5. Exclusion Criteria

Patients with COVID-19 who did not require ICU admission for invasive mechanical ventilation, patients with respiratory symptoms who tested negative for SARS-CoV-2 infection, and patients with chronic diseases of tumoral or viral origin, such as human immunodeficiency virus (HIV) infection, were excluded.

### 2.6. Data Source

Electronic medical records of all ICU-admitted patients who met the inclusion criteria were reviewed. Information on epidemiological, clinical, immunological, inflammatory, and hematological variables, as well as ICU mortality, was collected from the time of admission in order to determine survival and non-survival outcomes. Blood samples for hematological and inflammatory markers, including NLR and MPV, were collected exactly at 24 h following ICU admission. Samples were drawn via standard peripheral venous puncture or from an indwelling arterial line, according to the routine critical care protocols of each center, and were immediately processed using automated routine analyzers at each institution’s central laboratory. Hematological parameters, including neutrophil count, lymphocyte count, platelet count, and MPV, were measured using automated hematology analyzers according to local laboratory availability at each participating center. The analyzers used were the Sysmex XN-1000 automated hematology analyzer (Sysmex Corporation, Kobe, Japan) and the Beckman Coulter DxH 800 hematology analyzer (Beckman Coulter, Inc., Brea, CA, USA). NLR was calculated as the ratio of absolute neutrophil count to absolute lymphocyte count.

The primary outcome of the study was ICU mortality, defined as death occurring during the intensive care unit stay. All mortality analyses were based on this outcome.

Because this was a retrospective multicenter study conducted during different phases of the COVID-19 pandemic, ICU admission criteria, timing of intubation, ventilatory strategies, and pharmacological protocols were not fully standardized across centers. Clinical management followed the local institutional protocols and resource availability of each participating hospital.

### 2.7. Statistical Analysis

Data were coded and entered into a database accessible only to the principal investigator. Statistical analyses were performed using R statistical software, version 4.0.2 (R Foundation for Statistical Computing, Vienna, Austria). Missing data were handled using listwise deletion (complete-case analysis) for the respective statistical tests, and no imputation methods were applied. Descriptive statistics were presented in tables and graphs, including absolute and relative frequencies for qualitative variables and measures of central tendency and variability for quantitative variables.

The assumption of normality for quantitative variables was assessed using the Kolmogorov–Smirnov test. One-way analysis of variance (ANOVA) was used to compare altitude levels for normally distributed variables, whereas the Kruskal–Wallis test was applied for variables with non-normal distribution. Qualitative variables were analyzed according to altitude level using the chi-square test or Fisher’s exact test, as appropriate.

Multivariable logistic regression analysis was performed to explore factors associated with ICU mortality. The model was adjusted for age and sex. Because each altitude category corresponded to a single hospital, associations involving altitude should be interpreted as center-linked associations rather than as independent causal effects of altitude. Statistical significance was established at a *p*-value < 0.05.

To determine the cutoff points for NLR and MPV, a post hoc descriptive distribution-based analysis stratified by altitude level was performed. Specifically, the median and interquartile range (IQR) of the cohort were used to segment biomarker values and identify exploratory thresholds associated with ICU mortality within each altitude stratum. Receiver Operating Characteristic (ROC) curves, Area Under the Curve (AUC), sensitivity, specificity, positive predictive value, and negative predictive value were not calculated. Internal validation methods, such as bootstrapping or cross-validation, and external validation were not performed. Therefore, these altitude-stratified thresholds should be interpreted as exploratory and hypothesis-generating rather than validated prognostic cutoffs.

## 3. Results

### 3.1. Clinical Characteristics and Outcomes According to Altitude

When clinical characteristics were analyzed according to altitude level, significant differences were observed across groups (Table 1). The overall age was 54.44 years, and patients living at sea level and moderate altitude were significantly older than those living at high altitude (*p* < 0.001).

With regard to comorbidities, the prevalence of hypertension differed significantly according to altitude and was highest among patients living at sea level, followed by those at moderate altitude and high altitude (*p* < 0.001). Obesity also varied significantly across altitude levels, with the highest prevalence observed at moderate altitude, followed by high altitude and sea level (*p* < 0.001).

The APACHE II score at admission was significantly lower in patients living at moderate altitude than in those living at sea level or high altitude (*p* < 0.001). Mortality and length of hospital stay also differed significantly across altitude levels. Patients living at high altitude had the lowest mortality rate and the shortest hospital stay compared with those living at sea level and moderate altitude (*p* < 0.001).

### 3.2. Mechanical Ventilation and Laboratory Parameters Across Altitude Levels

Mechanical ventilation parameters also varied according to altitude level (Table 2). Tidal volume at 24 h was significantly lower in patients living at sea level than in those living at moderate and high altitude (*p* < 0.001). PEEP and plateau pressure also showed significant differences across altitude groups (*p* < 0.05 and *p* < 0.001, respectively), whereas no significant differences were observed for driving pressure or PaO_2_/FiO_2_.

Pulmonary compliance at 24 h was significantly lower in patients living at sea level than in those living at moderate and high altitude (*p* < 0.001). The duration of mechanical ventilation also differed significantly across groups, with the shortest duration observed in patients living at high altitude (*p* < 0.001).

Analytical and hematological parameters showed significant differences according to altitude level (Table 3). Ferritin levels were significantly lower in patients living at high altitude than in those living at sea level (*p* = 0.003). NLR values differed significantly across altitude levels and were highest in patients living at high altitude (*p* = 0.011). MPV values also differed significantly across groups, with the highest values observed at moderate altitude (*p* < 0.001). In addition, IL-6 levels were significantly higher at moderate altitude than at high altitude (*p* = 0.003). No significant differences were observed for D-dimer levels.

### 3.3. Factors Associated with Mortality and Altitude-Specific Prognostic Thresholds

In the age- and sex-adjusted logistic regression model, treatment at the sea-level and moderate-altitude centers was associated with higher odds of ICU mortality compared with treatment at the high-altitude center (Table 4). Patients treated at the sea-level center had 3.25-fold higher odds of ICU mortality than those treated at the high-altitude center (*p* < 0.001), whereas patients treated at the moderate-altitude center had 1.89-fold higher odds of ICU mortality (*p* = 0.022). However, because each altitude category corresponded to a single hospital, these associations should not be interpreted as independent effects of altitude.

Age > 55 years was also associated with mortality. Patients older than 55 years had 2.18-fold higher odds of death than those aged ≤55 years (*p* = 0.001). Male sex was likewise associated with mortality, with men showing 1.86-fold higher odds of death than women (*p* = 0.015).

Altitude-specific cut-off points for NLR and MPV were also associated with mortality (Table 5). At sea level, patients with NLR ≥ 12.50 had a significantly higher mortality rate than those with NLR < 12.50 (70.91% vs. 49.21%, *p* = 0.017), with an OR of 2.52. Likewise, MPV ≥ 8.80 fL was associated with markedly higher mortality compared with MPV < 8.80 fL (90.00% vs. 51.19%, *p* < 0.001), with an OR of 8.58.

At moderate altitude, patients with NLR ≥11.50 had higher mortality than those with NLR < 11.50 (60.71% vs. 41.82%, *p* = 0.046), corresponding to an OR of 2.15. Similarly, MPV ≥ 9.80 fL was associated with higher mortality than MPV < 9.80 fL (62.50% vs. 34.92%, *p* = 0.003), with an OR of 3.11.

At high altitude, patients with NLR ≥ 16.30 had significantly higher mortality than those with NLR < 16.30 (40.82% vs. 23.19%, *p* = 0.040), with an OR of 2.28. In the same group, MPV ≥ 9.00 fL was associated with higher mortality than MPV < 9.00 fL (50.00% vs. 24.39%, *p* = 0.010), with an OR of 3.10.

Overall, NLR and MPV showed altitude-stratified associations with ICU mortality among critically ill patients with COVID-19. However, these cutoff points were derived post hoc and were not independently validated; therefore, they should be considered exploratory rather than definitive prognostic thresholds.

## 4. Discussion

This retrospective multicenter study found that NLR and MPV values measured 24 h after ICU admission were associated with ICU mortality among mechanically ventilated patients with critical COVID-19. The identified altitude-stratified cutoff points should be interpreted as exploratory thresholds rather than validated prognostic standards, particularly because each altitude stratum corresponded to a different hospital and because the cutoffs were not derived or validated through ROC-based predictive modeling.

Obesity, a well-known proinflammatory state, was more prevalent at moderate altitude, followed by high altitude, whereas the population at sea level was the least obese but had the highest mortality. This finding is relevant and may suggest a complex interaction between obesity, altitude, and outcomes; however, this interpretation should be made cautiously, as our study was not designed to evaluate the mechanistic basis of a possible “obesity paradox” [13]. In a study conducted in the city of Quito, Ecuador, located at 2850 m above sea level, BMI was not associated with mortality in critically ill patients at high altitude [14].

Clinical severity at ICU admission was assessed using the APACHE II score (Acute Physiology and Chronic Health Evaluation II). High altitude was associated with greater disease severity, followed by sea level and moderate altitude, respectively. These results do not coincide with global reports, in which high altitude has been associated with lower severity and mortality [7]. This apparent discordance is noteworthy, since patients at high altitude showed higher APACHE II scores but lower mortality. Such findings suggest that the relationship between baseline severity, altitude, and outcomes may be more complex than expected and may also reflect differences in patient profiles, timing of ICU admission, or center-specific practices. This complexity may extend beyond acute critical illness. Altitude may not exert a linear or uniformly protective effect across all COVID-19 outcomes; rather, its impact may vary according to the stage of disease, the population studied, and the outcome assessed. This interpretation is consistent with prior evidence from Ecuador showing a higher frequency of self-reported Long COVID symptoms at high altitude, underscoring the need to interpret the role of altitude in COVID-19 with caution [15].

In COVID-19, increased IL-6 levels have been associated with progression to lung injury and hypoxemia, making it a reliable prognostic biomarker of disease severity [16,17]. In our study, IL-6 differed significantly between moderate- and high-altitude groups. However, this finding should be interpreted with caution, since information on this variable could not be obtained for patients at sea level, thereby limiting its comparability across all altitude strata and reducing its utility as a broadly extrapolatable biomarker in this cohort.

A systematic review showed that, in COVID-19 cases, non-survivors had higher serum ferritin levels compared with survivors [18]. Similarly, Bozkurt et al. reported in their study that ferritin level was the only significant predictor of disease severity (β = 0.487, t = 2.993, *p* = 0.004) [19]. High ferritin levels have been identified as an independent predictor of mortality [20,21]. Our study demonstrated higher ferritin levels in patients at sea level compared with those at moderate and high altitude, indicating a greater proinflammatory state in this group, which could have contributed to their higher mortality.

COVID-19 is characterized by a severe inflammatory state, particularly in its most critical presentation. The independent involvement of inflammatory cells such as neutrophils and lymphocytes has been shown to contribute to the high mortality of this virus in critically ill patients [22,23]. Inflammatory factors related to viral infection, such as interleukin-6, interleukin-8, and granulocyte colony-stimulating factor, may stimulate neutrophil production [24,25,26]. Conversely, systemic inflammation accelerates lymphocyte apoptosis, suppresses cellular immunity, reduces CD4+ T cells, and increases suppressor CD8+ T lymphocytes [27,28].

Based on this rationale, several recent studies have combined these inflammatory cells into a single index, obtained from the ratio between the absolute neutrophil count and the absolute lymphocyte count (NLR), whose behavior has more accurately highlighted its association with disease severity and mortality in COVID-19. A meta-analysis demonstrated that NLR at hospital admission predicts severity and mortality in patients with COVID-19, with higher NLR levels being associated with poor outcomes; however, this and other meta-analyses have not been able to validate an optimal cutoff point across different populations [29,30]. It is important to note that, in our study, patients were in an advanced stage of the disease, as samples were obtained after intubation. Previous studies have shown that patients with acute respiratory conditions tend to present higher neutrophil and monocyte counts, as well as an increased neutrophil-to-lymphocyte ratio (NLR). In contrast, patients with COVID-19 have been characterized by lower neutrophil, lymphocyte, and monocyte counts, together with a lower NLR [31].

A study conducted by Jingyuan et al. prospectively included 61 patients with confirmed COVID-19 treated at Beijing Ditan Hospital and observed that the incidence of critical illness was 9.1% among patients aged ≥50 years with an NLR < 3.13, while it was predicted that 50% of patients aged ≥50 years with an NLR ≥ 3.13 would develop critical disease [32]. For their part, Yildiz et al. observed in their study that an NLR cutoff value of 5.94 yielded a sensitivity of 62% and a specificity of 64% [33]. In contrast, Tadesse et al. identified an NLR cutoff value of 9.47 as the optimal threshold for predicting mortality, with a sensitivity of 88.7% and a specificity of 95.4% [34]. Another retrospective cohort study evaluating the clinical course and mortality of COVID-19, conducted in 223 adult patients admitted to the ICU and receiving invasive mechanical ventilation in the city of Quito, Ecuador (2850 m above sea level), identified an NLR cutoff value ≥22 at 24 h as predictive of mortality [8]. This cutoff contrasts markedly with that reported by Jingyuan et al., although these studies are not directly comparable because they differed in clinical setting, disease severity, timing of biomarker assessment, and outcome definition. Differences in geographic altitude between the studied populations may partly explain this discrepancy, but other factors, including population characteristics and case mix, are also likely to contribute.

The results of our study suggest that NLR may be associated with ICU mortality in critically ill patients across different altitude strata. However, the cutoff identified in our cohort differed from that reported in a previous study from Quito [8], and the threshold observed at sea level was higher than that reported by Jingyuan et al. These differences may be explained by variations in disease severity, timing of biomarker assessment, population characteristics, case mix, clinical management, and study setting. Therefore, direct comparisons across studies should be made cautiously.

**Figure 1:**
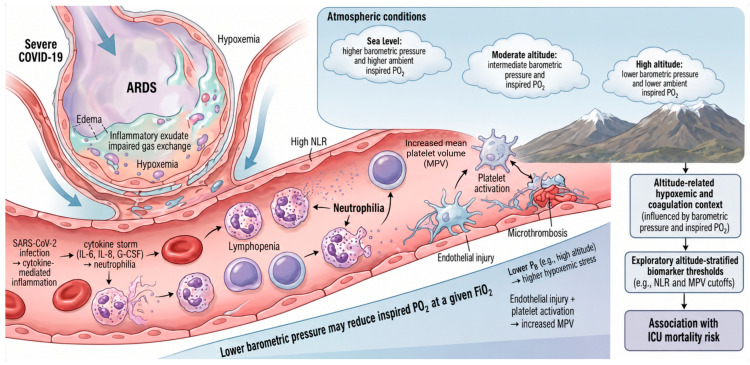
Conceptual summary linking systemic inflammation, blood-cell alterations, platelet activation, endothelial injury, and altitude-related oxygen physiology in critically ill patients with COVID-19. The figure summarizes a plausible conceptual pathway by which SARS-CoV-2 infection may trigger pulmonary inflammation, acute respiratory distress syndrome (ARDS), hypoxemia, and systemic cytokine activation involving mediators such as IL-6, IL-8, and G-CSF. This inflammatory response may promote neutrophilia, lymphocyte apoptosis, and lymphopenia, resulting in an elevated neutrophil-to-lymphocyte ratio (NLR), while also contributing to platelet activation and increased mean platelet volume (MPV). Concurrent endothelial injury and platelet activation may favor microthrombosis, impaired gas exchange, organ dysfunction, and higher observed ICU mortality risk. The diagram also integrates the potential influence of geographic altitude, illustrating how lower barometric pressure and reduced inspired PO_2_ at a given FiO_2_ may modify the hypoxemic context and the interpretation of inflammatory and hematological biomarkers. This framework should be interpreted as a conceptual summary based on biological plausibility and observed associations in this retrospective cohort; the proposed pathways and biomarker thresholds were not directly tested or validated.

The proinflammatory condition of COVID-19 induces prothrombotic states and endotheliitis; therefore, it has been proposed that increased platelet size and activation may be associated with greater disease severity and mortality [35,36,37,38,39]. Sertbas et al. conducted a multicenter study with nearly 10,000 patients, which demonstrated that an MPV value >10.05 femtoliters (fL) adequately predicts mortality in patients with COVID-19 (OR 5.15). However, the external validity of these results is limited, as the study population was restricted to sea-level patients [40]. In our study, MPV was also associated with ICU mortality, with different exploratory cutoff points observed across altitude strata. Nevertheless, these thresholds were derived from the same cohort and were not validated internally or externally. Therefore, MPV should be considered a potentially informative biomarker in this context, but not yet a validated prognostic tool for routine clinical decision-making. Figure 1 summarizes the proposed pathophysiological framework linking systemic inflammation, hematologic alterations, platelet activation, endothelial injury, and altitude-related oxygen physiology with mortality in critically ill patients with COVID-19. It is important to note that the downregulation of ACE2 leads to a compensatory overproduction of angiotensin II, which stimulates the angiotensin II type 1a receptor (AT1a), thereby increasing pulmonary vascular permeability and exacerbating lung pathology [41].

At the beginning of the pandemic, as COVID-19 was a novel and unprecedented infection, the clinical management of patients was challenging. Initially, upon ICU admission, helmet continuous positive airway pressure (CPAP) was used as the primary mode of ventilatory support [42]. Subsequently, it was reported that relatively lower mortality was observed among patients who underwent early intubation, received high-flow nasal cannula therapy, and avoided CPAP [42].

As knowledge about COVID-19 evolved, an increased use of non-invasive respiratory support, a shorter duration of invasive mechanical ventilation (IMV) among ICU patients, and greater use of prone positioning and corticosteroids were observed. These changes contributed to a reduction in the number of patients requiring IMV, as well as shorter ICU and hospital lengths of stay [43].

Regarding pharmacological treatments, tocilizumab has been reported to reduce the 30-day mortality rate. However, previous studies have shown that the use of monoclonal antibodies does not result in differences in therapeutic response between obese and non-obese patients with COVID-19 [44,45]. In addition, molnupiravir has been shown to be effective and well tolerated. In their study, Pontolillo et al. observed that molnupiravir led to symptom resolution within one week and viral load negativization within ten days, and none of the patients who attended clinical follow-up experienced viral rebound after treatment with molnupiravir [46]. On the other hand, with respect to corticosteroid therapy, deflazacort has been associated with a lower incidence of hospitalization due to glycemic decompensation compared with standard treatment using dexamethasone or methylprednisolone in outpatients with confirmed SARS-CoV-2 infection [47]. 

An important aspect that should be considered when interpreting these findings is that altitude level was structurally linked to hospital center, since each altitude stratum corresponded to a different institution. Therefore, the observed differences cannot be attributed exclusively to altitude. They may also reflect differences in ICU admission criteria, timing of intubation, ventilatory strategies, patient referral patterns, resource availability, pharmacological treatments, and local standards of care. This limitation is central to the interpretation of the study and prevents the attribution of an independent causal effect to altitude.

### Limitations

This study has several limitations. Its retrospective design and reliance on secondary clinical records may have introduced information bias and limited the availability of relevant variables. The study included only critically ill patients with COVID-19 requiring invasive mechanical ventilation, which limits extrapolation to patients with milder disease or earlier stages of care. In addition, each altitude stratum corresponded to a single hospital, making it impossible to fully separate the effect of geographic altitude from center-specific factors such as ICU admission criteria, timing of intubation, ventilatory practices, therapeutic protocols, resource availability, and referral patterns. Not all potential confounders, including detailed treatment variables, duration of illness before ICU admission, vaccination status, and some laboratory biomarkers, were consistently available or incorporated into the multivariable model. IL-6 was not available at the sea-level center, limiting comparisons across all three altitude groups. Furthermore, NLR and MPV were not tested as continuous variables or included as independent predictors in the multivariable model. Finally, the NLR and MPV cutoff points were derived from the same cohort using a post hoc exploratory distribution-based approach and were not validated using ROC analysis, bootstrapping, cross-validation, or an external cohort. Therefore, these thresholds should be considered hypothesis-generating rather than definitive clinical standards.

## 5. Conclusions

In mechanically ventilated patients with critical COVID-19, NLR and MPV values above post hoc, altitude-stratified cutoff points were associated with higher ICU mortality. However, these thresholds were not derived through ROC analysis and were not internally or externally validated. In addition, because each altitude stratum corresponded to a different hospital, the observed differences may reflect center-specific factors as well as altitude-related physiology. Therefore, these findings should be interpreted as exploratory and hypothesis-generating. Future studies should include multiple hospitals at each altitude level, incorporate standardized clinical and therapeutic variables, and apply internal and external validation methods before NLR and MPV thresholds can be considered for routine risk stratification.

## Figures and Tables

**Table 1:** Relationship between clinical characteristics and altitude level.

Clinical Characteristics		Total	Altitude Level			*p*-Value
			Sea Level	Moderate	High	
Age (mean ± SD), years		54.44 (12.68)	57.95 (13.62)	54.85 (10.66)	50.55 (12.58)	<0.001 ^1^
Sex (*n* (%))	Male	258 (71.27)	77 (64.17)	94 (77.69)	87 (71.9)	0.067 ^2^
	Female	104 (28.73)	43 (35.83)	27 (22.31)	34 (28.1)	
Diabetes Mellitus (*n* (%))	Yes	72 (20.06)	29 (24.17)	25 (21.01)	18 (15)	0.197 ^2^
	No	287 (79.94)	91 (75.83)	94 (78.99)	102 (85)	
Hypertension (*n* (%))	Yes	105 (29.09)	55 (45.83)	31 (25.83)	19 (15.7)	<0.001 ^2^
	No	256 (70.91)	65 (54.17)	89 (74.17)	102 (84.3)	
Obesity (*n* (%))	Yes	94 (25.97)	3 (2.5)	53 (43.8)	38 (31.4)	<0.001 ^2^
	No	268 (74.03)	117 (97.5)	68 (56.2)	83 (68.6)	
APACHE II score at admission (median [IQR])		13 (8–18)	15 (11–19)	8 (6–12)	16 (12–20)	<0.001 ^1^
Mortality (*n* (%))		163 (45.53)	70 (59.32)	57 (47.5)	36 (30)	<0.001 ^2^
Length of hospital stay (median [IQR])		14 (8–21)	15 (8–22)	18 (12–24)	10 (7–16)	<0.001 ^1^

Notes: SD = standard deviation; IQR = interquartile range. ^1^ Kruskal–Wallis test; ^2^ Chi-square test.

**Table 2:** Relationship between mechanical ventilation parameters and altitude level.

Mechanical Ventilation Parameters by Altitude Level	Altitude			*p*-Value
	Sea Level *n* = 120	Moderate *n* = 121	High *n* = 121	
Tidal volume at 24 h (median [IQR]), mL/kg	380 [360–400]	450 [400–500]	410 [390–460]	<0.001 ^1^
PEEP at 24 h (median [IQR]), cmH_2_O	11 [8.75–12.25]	10 [9–12]	10 [8–12]	<0.040 ^1^
Plateau pressure at 24 h (median [IQR]), cmH_2_O	26 [24–29]	24 [20.52–26.21]	23 [22–25]	<0.001 ^1^
Driving pressure at 24 h (median [IQR]), cmH_2_O	14 [11–18]	13 [10.88–15.31]	14 [12–15]	0.268 ^1^
PaO_2_/FiO_2_ at 24 h (median [IQR]), mmHg	115 [87.81166.17]	133 [97.25–172]	151 [117–167]	0.168 ^1^
Compliance at 24 h (mean ± SD), mL/cmH_2_O	26.78 (13.01)	35.67 (9.02)	33.49 (10.22)	<0.001 ^2^
Days on mechanical ventilation (median [IQR])	11 [6–18.5]	12 [10–19]	7 [4–12]	<0.001 ^1^

Notes: SD = standard deviation; IQR = interquartile range; ^1^ Kruskal–Wallis test; ^2^ ANOVA test.

**Table 3:** Relationship between analytical and cytometry parameters and altitude level.

Analytical and Cytometry Parameters	Altitude			*p*-Value
	Sea Level *n* = 120	Moderate *n* = 121	High *n* = 121	
D-dimer at 24 h (median [IQR]) ng/mL	2000 [400–6500]	930 [390–1510]	1059 [747–1901]	0.114 ^1^
Ferritin at 24 h (median [IQR]) ng/mL	1500 [1008–2000]	1478 [617.4–2000]	1158 [664–1545]	0.003 ^1^
NLR at 24 h (median [IQR])	12 [7.17–17.8]	13 [11.35–13.70]	15 [9.71–23.72]	0.011 ^1^
MPV at 24 h (median [IQR]) fL	8.35 [7.9–8.8]	9.80 [9.1–10.5]	8.80 [8.5–9.5]	<0.001 ^1^
IL-6 (median [IQR]) pg/mL		93 [27.3–176.5]	25 [11.2–78.2]	0.003 ^2^

Note: IQR = Interquartile Range; NLR = neutrophil-to-lymphocyte ratio; MPV = mean platelet volume; IL-6 = interleukin-6. ^1^ Kruskal–Wallis test; ^2^ Mann–Whitney test.

**Table 4:** Age- and sex-adjusted logistic regression model for ICU mortality in critically ill COVID-19 patients.

Variables	β	*p*-Value	OR	95% CI for OR	
				Lower	Upper
Altitude					
Sea level	1.18	<0.001 *	3.25 **	1.86	5.67
Moderate altitude	0.64	0.022 *	1.89 **	1.10	3.26
Age > 55 year	0.78	0.001 *	2.18 **	1.40	3.41
Male	0.62	0.015 *	1.86 **	1.13	3.07

Note: OR = Odds Ratio; CI = Confidence Interval; * Statistically significant association with ICU mortality; ** Association should not be interpreted as an independent causal effect of altitude because each altitude stratum corresponded to a single hospital.

**Table 5:** Relationship between cut-off points and mortality according to altitude type.

Altitude/Cut-Off Points		Discharge Status		*p*-Value	Unadjusted OR (95% CI)
		Non Survivor *n* (%)	Survivor *n* (%)		
Sea level					
NLR at 24 h	≥12.50	39 (70.91)	16 (29.09)	0.017 *	2.52 ** (1.17–5.40)
	<12.50	31 (49.21)	32 (50.79)		
MPV at 24 h	≥8.80 fL	27 (90.00)	3 (10)	<0.001 *	8.58 ** (2.42–30.47)
	<8.80 fL	43 (51.19)	41 (48.81)		
Moderate altitude					
NLR at 24 h	≥11.50	34 (60.71)	22 (39.29)	0.046 *	2.15 ** (1.01–4.59)
	<11.50	23 (41.82)	32 (58.18)		
MPV at 24 h	≥9.80 fL	35 (62.5)	21 (37.5)	0.003 *	3.11 ** (1.47–6.60)
	<9.80 fL	22 (34.92)	41 (65.08)		
High altitude					
NLR at 24 h	≥16.30	20 (40.82)	29 (59.18)	0.040 *	2.28 ** (1.03–5.08)
	<16.30	16 (23.19)	53 (76.81)		
MPV at 24 h	≥9.00 fL	15 (50)	15 (50)	0.010 *	3.10 ** (1.29–7.44)
	<9.00 fL	20 (24.39)	62 (75.61)		

Note: * Statistically significant differences in mortality; ** Cut-off point considered a risk factor; analysis based on the Chi-square test; OR = Unadjusted Odds Ratio.

## Data Availability

The original contributions presented in this study are included in the article. Further inquiries can be directed to the corresponding author.

## Abbreviations

NLR	neutrophil-to-lymphocyte ratio
ICU	intensive care unit
ARDS	acute respiratory distress syndrome
MPV	mean platelet volume
masl	meters above sea level
HIV	human immunodeficiency virus
SD	standard deviation
IQR	Interquartile Range
OR	Odds Ratio
CI	Confidence Interval
